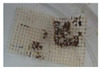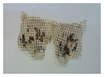# Controlled Release, Disintegration, Antioxidant, and Antimicrobial Properties of Poly (Lactic Acid)/Thymol/Nanoclay Composites

**DOI:** 10.3390/polym12091878

**Published:** 2020-08-20

**Authors:** Marina Ramos, Elena Fortunati, Ana Beltrán, Mercedes Peltzer, Francesco Cristofaro, Livia Visai, Artur J.M. Valente, Alfonso Jiménez, José María Kenny, María Carmen Garrigós

**Affiliations:** 1 Nutrition & Food Sciences, Department of Analytical Chemistry, University of Alicante, 03080 Alicante, Spain; ana.beltran@ua.es (A.B.); alfjimenez@ua.es (A.J.); mc.garrigos@ua.es (M.C.G.); 2 Civil Environmental Engineering Department, University of Perugia, UdR INSTM, Strada di Pentima 4, 05100 Terni, Italy; elenafortunati@gmail.com (E.F.); jose.kenny@unipg.it (J.M.K.); 3 Departamento de Ciencia y Tecnología, Universidad Nacional de Quilmes, Bernal, Buenos Aires B1876BXD, Argentina; mercedes.peltzer@unq.edu.ar; 4 Consejo Nacional de Investigaciones Científicas y Técnicas (CONICET), Ciudad Autónoma de Buenos Aires (CABA), Buenos Aires C1425FQB, Argentina; 5 Department of Molecular Medicine, Center for Health Technologies (C.H.T.), UdR INSTM, University of Pavia, 27100 Pavia, Italy; francesco.cristofaro01@universitadipavia.it (F.C.); livia.visai@unipv.it (L.V.); 6 Department of Occupational Medicine, Toxicology and Environmental Risks, Istituti Clinici Scientifici (ICS) Maugeri, Società Benefit S.p.A IRCCS, 27100 Pavia, Italy; 7 Department of Chemistry, University of Coimbra, CQC, 3004-535 Coimbra, Portugal; avalente@ci.uc.pt

**Keywords:** PLA, nanocomposites, functional properties, thymol, migration, films

## Abstract

Nano-biocomposite films based on poly (lactic acid) (PLA) were prepared by adding thymol (8 wt.%) and a commercial montmorillonite (D43B) at different concentrations (2.5 and 5 wt.%). The antioxidant, antimicrobial, and disintegration properties of all films were determined. A kinetic study was carried out to evaluate the thymol release from the polymer matrix into ethanol 10% (*v*/*v*) as food simulant. The nanostructured networks formed in binary and ternary systems were of interest in controlling the release of thymol into the food simulant. The results indicated that the diffusion of thymol through the PLA matrix was influenced by the presence of the nanoclay. Disintegration tests demonstrated that the incorporation of both additives promoted the breakdown of the polymer matrix due to the presence of the reactive hydroxyl group in the thymol structure and ammonium groups in D43B. Active films containing thymol and D43B efficiently enhanced the antioxidant activity (inhibition values higher than 77%) of the nano-biocomposites. Finally, the addition of 8 wt.% thymol and 2.5 wt.% D43B significantly increased the antibacterial activity against *Escherichia coli* and *Staphylococcus aureus 8325-4,* resulting in a clear advantage to improve the shelf-life of perishable packaged food.

## 1. Introduction

The use of biopolymers in packaging applications is considered a suitable alternative to the petrochemical counterparts contributing to the limitation of the environmental problems caused by the accumulation of plastic waste [1]. Among the different bio-based polymers that can be used for such purpose, poly (lactic acid) or PLA is a biodegradable polyester obtained from 100% renewable resources. It is also a highly versatile material with many commercial applications in the textile, medical, automotive and packaging sectors. PLA shows some desirable properties such as its inherent biodegradability, biocompatibility, stiffness, high strength, thermo-plasticity, and high transparency [2]. However, PLA-based films show some significant drawbacks which limit their performance in processing and the final use, such as poor barrier properties to gases, low thermal and mechanical resistance, slow crystallization and brittleness [3].

These shortcomings can be overcome by the development of nano-biocomposites based on PLA reinforced with nanoparticles, which has been introduced as a promising option [4]. Souza et al. [5] assumed that the incorporation of nanoclays into PLA-based formulations is a promising alternative to improve their barrier and mechanical properties without altering the transparency and compostability of the final material. Among the different nanoparticles, an interesting approach to improve PLA properties is the reinforcement with nanoclays such as montmorillonites, resulting in the development of PLA nanocomposites [6]. The use of this type of nanomaterials in food packaging applications can also modify the internal atmosphere due to the modification of barrier properties, thereby delaying ripening and extending the shelf-life in packaged foods [7].

The development of new nanocomposites with unique characteristics is desirable, especially for the food industry due to their full range of applications [8]. The satisfaction of consumers’ requirements for healthier and highly nutritional foods, as well as the increase in the need for long shelf-life for fresh food, have resulted in a high interest in the use of functional packaging systems with a controlled release of active substances embedded in eco-friendly plastic materials [9]. Active packaging systems incorporate agents with specific functionalities into the polymer matrix [10,11], reacting with or releasing specific components to increase food quality, taking advantage of these interactions [2]. Low density polyethylene (LDPE) was combined with thymol, eugenol, and carvacrol embedded in montmorillonite or halloysite to produce active nanocomposite films [12]. These formulations improved the main properties of LDPE as well as food quality by the increase in antimicrobial and antioxidant performance, and enhanced barrier properties. Montmorillonite was also used in alginate-based films with lemon essential oil to obtain antibacterial and antifungal films [13]. Consequently, the combination of natural compounds extracted from plant species with antimicrobial and antioxidant properties with bio-based polymers to enhance their functional properties and to extend food shelf-life is a promising strategy to be applied in the food sector [14,15,16,17,18,19,20]. In particular, thymol which is the main phenolic compound present in thyme and oregano essential oils has been used due to its antimicrobial and antioxidant properties that make it useful to be incorporated as an active compound in packaging formulations [21,22,23,24]. To the best of our knowledge, the combination of thymol and montmorillonite in PLA-based films to obtain active nanocomposites with both antimicrobial and antioxidant properties has not been extensively studied. This combination can be considered an interesting approach in food science to reduce the oxidative and microbial deterioration of food products to increase food quality, safety and shelf-life while maintaining the material properties. Several works on PLA nanocomposites have been already published [25,26,27,28,29], but the present work could contribute to fill a gap in the development of new sustainable nanocomposite films with combined antioxidant and antibacterial performance by controlling the release of the active agent. In addition, it is important to validate the potential use of these materials in active packaging applications.

In a previous work, these novel nano-biocomposite films based on PLA with D43B and thymol for active packaging were successfully characterized in their physicochemical properties [30]. However, the evaluation of the controlled release of the active agent into a specific food-grade simulant (ethanol 10% *v*/*v*), disintegration, antioxidant and antimicrobial properties of the obtained nano-biocomposite films has not been published yet. Therefore, the aim of this study is the evaluation of the functional and compostability properties of these films for their potential use as active food packaging materials, demonstrating also the effective release of thymol from the active nano-biocomposite PLA films.

## 2. Materials and Methods

### 2.1. Materials

Commercial PLA-4060D (Tg = 58 °C, 11–13 wt.% D-isomer) was supplied in pellets by Natureworks Co., (Minnetonka, MN, USA). Thymol (99.5%), 2,2-Diphenyl-1-picrylhydrazyl (DPPH, 95%), methanol and ethanol (High-performance liquid chromatography, HPLC grade) were supplied by Sigma-Aldrich (Madrid, Spain). The commercial nanoclay used was Dellite^®^43B (D43B) (Laviosa Chimica Mineraria S.p.A. Livorno, Italy), a dimethyl-benzyldihydrogenated tallow ammonium modified montmorillonite with a cation exchange capacity (CEC) of 95 meq/100 g clay, a bulk density of 0.40 g cm^−3^ and a particle size distribution between 7–9 µm.

### 2.2. Nano-Biocomposite Films Preparation

PLA-based nano-biocomposite films incorporated with D43B (as nanofiller) and thymol (as active agent) were developed by melt blending in a Haake Polylab QC mixer (ThermoFischer Scientific, Walham, MA, USA) at 160 °C as already described in a previous work [30]. Five formulations were obtained by combining thymol (8 wt.%) and D43B at two different loadings (2.5 and 5 wt.%) in PLA matrices. Neat PLA was used as control. Homogenous and transparent films were obtained by compression-molding as described in [29]. The final mean film thickness was 190 ± 5 μm measured with a Digimatic Micrometer Series 293 MDC-Lite (Mitutoyo, Japan) at five random positions.

In our previous work [29], it was concluded that the addition of thymol did not significantly affect the thermal stability of PLA, but some decrease (around 15%) in elastic modulus was observed due to the slight plasticizing effect induced by the active additive. The incorporation of D43B and thymol to PLA did not result in an apparent enhancement in oxygen barrier properties, but the tensile behavior was improved due to the intercalation and partial exfoliation of nanoparticles through the polymer matrix, as observed by X-Ray diffraction (XRD). Moreover, the intrinsic transparency of PLA was not affected by the addition of both components and most of the thymol initially added to PLA (around 70–75%) remained in the nanocomposites after processing, ensuring the potential applicability of these films as active systems.

### 2.3. Migration and Mathematical Diffusion Analysis

Migration tests for thymol released from the different nano-biocomposite films were performed, in triplicate, into ethanol 10% (*v*/*v*) as food simulant following the legislation for food contact materials EU/10/2011 [31] and the European Standard EN 13130-2005 [32]. Double-sided, total immersion migration tests were performed with films (12 cm^2^). 20 mL of food simulant were put in contact with the nano-biocomposite films (area-to-volume ratio around 6 dm^2^ L^−1^) at 40 °C in an oven (J.P. Selecta, Barcelona, Spain) for 10 days. A blank sample (pure simulant) was also studied.

On the other hand, the release mechanism of thymol from PLA nano-biocomposite films was also studied over time by modelling the obtained results for 15 days in ethanol 10% (*v*/*v*). Different approaches have been applied to assess the migration of additives and contaminants from food packaging films: (i) a quantitative assessment of *M_F,∞_* to analyze the diffusion process by using Equation (1) to fit the experimental data,

(1) MF,tMP,o=(MF,∞MP,0)·(1−e−k’t)

where *M*_P,0_ is the initial amount of thymol inside the polymeric matrix, determined by using the HPLC-UV method optimized in our previous study [30]; *k’* is a constant; and *M_F,t_* is the mean of thymol released mass to the food simulant at a defined time; (ii) a Fick’s approach (diffusion-controlled systems) to assess the migration of thymol from films by using mathematical modelling (Equation (2)). This differential equation provides a general description of the migration of an additive or contaminant from an amorphous polymer packaging film:

(2) $$\frac{\partial C}{\partial t}=\frac{\partial }{\partial x}\left(D\frac{\partial C}{\partial x}\right)$$

where *D* is the diffusion coefficient; *c* is the concentration of the released species; *t* is time (s); and *x* is the space coordinate.

Apparent partition coefficients (*α*) can be calculated by Equation (3) from the values obtained for *M_F,∞_/M_P,0_* by fitting Equation (1),

(3) MF,∞MP,0=1(1+α)

where *α* is defined as:

(4) α=VFKP,FVP

where *V_P_* and *V_F_* are the volumes of the polymer sample (*P*) and food simulant (*F*), respectively; and *K_P,F_* is the partition coefficient of thymol in the system between the samples and the solution, which can be assumed as constant at low concentrations and also for its relative solubility at the equilibrium between PLA and the food simulant [33].

Crank solved Equation (2) and formulated initial and boundary conditions, from which Equation (5) was obtained as a solution. This equation was applied for a plane sheet of thickness *l*, and the initial condition for testing −*l*/2 < *x* < *l*/2, considering constant the thymol’s concentration released, a boundary condition of a partition coefficient between both phases and for one-dimensional diffusion of thymol in a limited volume solution [34],

(5) $$\frac{M_{F,t}}{M_{F,\infty }}=1-\overset{\infty }{\underset{n=1}{\sum }}\frac{2\alpha \left(1+\alpha \right)}{{1+\alpha +\alpha }^{2}q_{n}^{2}}\exp\left[\frac{{-Dq}_{n}^{2}t}{l^{2}}\right]$$

where *q_n_* is the non-zero positive roots of *tanq_n_ = −α q_n_* and *l* is the polymer matrix half-thickness.

The root mean square error (RMSE) was used to estimate the quality of the model fitting and it was calculated by following Equation (6),

(6) RMSE=[∑i=1n(yi−y^i)2n]12

where *y_i_* and *ŷ_i_* are, respectively, the experimental and predicted residual value; and *n* is the number of experimental points per migration curve.

High performance liquid chromatography coupled to an ultraviolet spectrophotometry detector (HPLC-UV) was used to determine the amount of thymol released from films at different migration times. An Agilent 1260 Infinity-HPLC Diode Array Detector (DAD) (Agilent, Santa Clara, CA, USA) and an Agilent Eclipse Plus C18 (100 mm × 4.6 mm × 3.5 μm) column were used. The mobile phase was composed of acetonitrile/water (40:60) at 1 mL min^−1^ flow rate. 20 μL of the extracts were injected and thymol was detected at λ = 274 nm. Analyses were performed in triplicate. Calibration standards were run at different concentrations between 12.5 and 780 mg kg^−1^ directly obtained from a stock solution (1000 mg kg^−1^) using appropriately diluted standards of thymol in ethanol 10% (*v*/*v*). The method was validated by the calculation of the main analytical parameters affecting the determination of thymol in the studied food simulant. Limit of detection (LOD) and limit of quantification (LOQ) values were determined by using regression parameters from the calibration curve (3 S_y/x/a_ and 10 *S_y/x/a_*, respectively; where *S_y/x_* is the standard deviation of the residues and a is the slope of the calibration curve) obtaining values of 0.29 mg_Thymol_ kg^−1^ and 0.96 mg_Thymol_ kg^−1^, respectively. An excellent linearity was obtained with a determination coefficient R^2^ of 0.9994.

### 2.4. Determination of Antioxidant Activity

The antioxidant activity of the obtained migration extracts was evaluated to study the effect of thymol released from the nano-biocomposite films into ethanol (10%, *v*/*v*) after 10 days by using the spectrophotometric method based on the formation of the stable radical 2,2-diphenyl-1-picrylhydrazyl (DPPH), as described elsewhere [30].

### 2.5. Bacterial Strains, Culture Conditions and Antibacterial Activity

*Escherichia coli* (*E. coli*) was provided by the Zooprofilattico Institute of Pavia (Italy) as an isolate strain whereas *Staphylococcus aureus 8325-4* (*S. aureus 8325-4*) was obtained from Timothy J. Foster (Department of Microbiology, Dublin, Ireland). *E. coli* and *S. aureus 8325-4* were grown overnight under aerobic conditions at 37 °C in Luria Bertani Broth (LB) and Brian Heart Infusion (BHI) (Difco Laboratories Inc., Detroit, MI, USA), respectively. The final density of these cultures was established at 1 × 10^10^ cells mL^−1^, determined by comparison of the optical density at 600 nm.

The evaluation of the antibacterial activity of the films was performed in 100 µL of a diluted cell suspension (1 × 10^3^ cells mL^−1^) of *E. coli* and *S. aureus 8325-4* maintained overnight. Bacterial strains were added to 3 × 3 mm^2^ samples, seeded at the bottom of a 96-well tissue culture plate and incubated at three different temperatures: 4 °C, 24 °C and 37 °C for 3 h and 24 h, respectively. These temperatures were selected to simulate refrigeration conditions (4 °C), ambient temperature (24 °C) and the usual incubation temperature of microbiological tests (37 °C). Regarding time, 3 h simulated a fast contact between the polymer sample and the bacteria whereas 24 h is the usual time used to evaluate the microbiological growth in antimicrobial tests.

Furthermore, 96-well flat-bottom sterile polystyrene culture plates were used as control under the same experimental conditions. At the end of each incubation period, bacterial suspensions were serially diluted and, after the incubation for 24/48 h at 37 °C, cell survival was expressed as the percentage of colony-forming unit, CFU, of bacterial growth on active nano-biocomposite films compared to those obtained for the neat PLA film.

### 2.6. Study of Disintegrability Under Composting Conditions

Disintegration tests were performed by following the ISO 20,200 Standard [35]. Film samples for disintegration tests were cut in pieces (20 × 20 mm^2^) and they were buried at 5 cm depth in perforated boxes with a solid synthetic bio-waste and incubated at 58 °C for 35 days. Different time intervals were selected to recover samples from their burial, and they were further tested after 0, 2, 4, 7, 10, 14, 21, 28 and 35 days. The degree of disintegration (%) was calculated by normalizing the sample weight at different stages of incubation to the initial weight, following Equation (7):

(7) $$\mathrm{Disintegrability}\;\left(\%\right)=\frac{W_{i}{-W}_{t}}{W_{i}}\cdot 100$$

where *W_i_* is the initial dry plastic weight, and *W_t_* is the dry plastic weight at the end of the test.

The degradation of the chemical structure of the nano-biocomposite films during the disintegration tests was estimated by comparing Fourier transform infrared spectroscopy (FTIR) spectra and differential scanning calorimetry (DSC) thermograms at different test stages. Furthermore, the changes in the visual appearance of the samples were also evaluated.

DSC analysis of samples at different disintegration times was carried out from −25 to 180 °C at a heating rate of 10 °C min^−1^ by using a DSC Mettler Toledo 822/e equipment (Schwarzenbach, Switzerland) working under nitrogen atmosphere (50 mL min^−1^). FTIR spectra of degraded samples were recorded by using a Jasco FT-IR 615 spectrometer (Jasco Inc., Easton, MD, USA) in attenuated total reflection (ATR) mode at 400–4000 cm^−1^ range.

### 2.7. Statistical Analysis

Statistical analysis of results was performed by using the SPSS commercial software (Version 15.0, Chicago, IL, USA). A one-way analysis of variance (ANOVA) was carried out. Differences between means were assessed based on confidence intervals using the Tukey test at a *p* < 0.05 significance level.

## 3. Results

### 3.1. Migration Test and Antioxidant Activity of the Active PLA Nano-Biocomposite Films

The use of nanofillers in active packaging systems has revealed their ability in controlling the release of active additives from polymer matrices, at suitable rates, improving their action [7]. In this work, the effect of the nanoclay in controlling the release kinetics of thymol from the PLA nano-biocomposite films to ethanol 10% (*v*/*v*) at 40 °C was evaluated. The amounts of thymol migrated in the simulant after 10 days were 285.0 ± 3.3, 275.5 ± 13.8 and 235.3 ± 19.4 mg_thymol_ kg^−1^_food simulant_ for PLA/T, PLA/T/D43B2.5 and PLA/T/D43B5, respectively. These results indicate that the formulation with the highest amount of D43B (PLA/T/D43B5) showed the lowest migration rate, retaining a higher amount of thymol in the polymer structure after 10 days. This behavior is in agreement with previously reported studies [36] indicating that the tortuosity effect imposed by the presence of D43B in the diffusion of the active compound through the matrix plays an important role in the release of thymol from PLA-based nano-biocomposite films. Campos-Requena et al. [37] observed that the intercalated morphology of organic modified montmorillonite/LDPE nanocomposite films had some influence on the release rate of thymol resulting in a decrease by approximately 15%, providing a controlled release material.

The antioxidant capacity of thymol released from the developed nano-biocomposite films was measured by analyzing the extracts obtained after 10 days by using the DPPH method. The inhibition values obtained were 77.8 ± 0.1, 77.0 ± 0.4, and 77.8 ± 0.8% for PLA/T, PLA/T/D43B2.5, and PLA/T/D43B5, respectively, showing the efficient antioxidant performance of thymol added in PLA-based nano-biocomposite films. This high antioxidant activity can be attributed to the capability of the thymol phenolic hydroxyl groups to convert the phenolic oxygen anion in an alkaline environment [38]. Moreover, thymol has been reported to be a better antioxidant in lipids than its isomer carvacrol due to the more significant steric hindrance of the phenolic group [39].

#### Mathematical Modelling of Thymol Released from PLA Nano-Biocomposite Films

Release studies are necessary and highly relevant when active substances with antimicrobial or antioxidant properties are incorporated into packaging materials to enhance the safety and quality of food during long storage. The kinetics of these processes is indicative of the transport phenomena inside the matrix and gives an accurate estimation of the effective release rate of the active compounds. In this process, the migrant starts to diffuse through the amorphous portion of the polymer matrix toward the interface with the driving force of the concentration gradient. The migrant’s concentration is partitioned between the two media until its potential chemical values, in both the polymer and the food, reach equilibrium. Thus, the release is the result of diffusion, dissolution and equilibrium processes, which are often described by the Fick’s second law [40].

**Figure 1:**
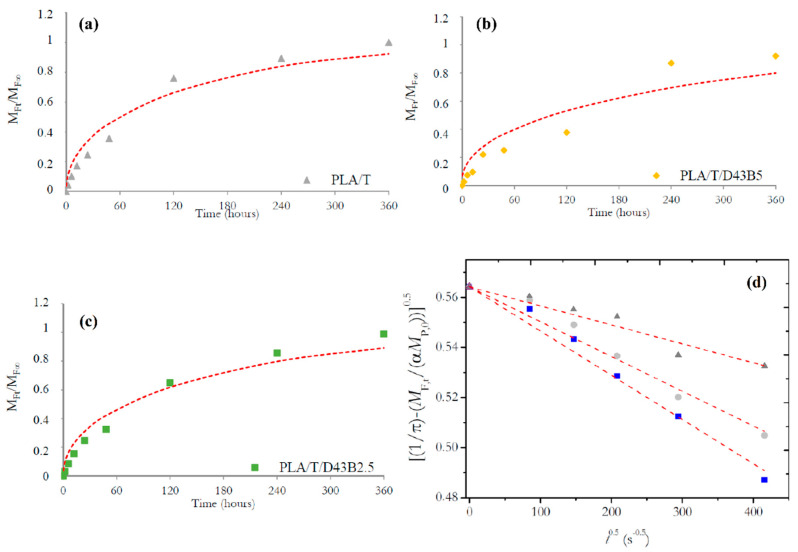
Normalized release of thymol from different polymer matrices: (**a**) PLA/T, (**b**) PLA/T/D43B2.5, and (**c**) PLA/T/D43B5; (**d**) plots of ${\left[\;\frac{1}{\pi }-\frac{1}{\alpha }\times \frac{M_{F,t}}{M_{F,0}}\right]}^{0.5}$ versus $t^{0.5}$ for the migration of thymol from: PLA/T (■), PLA/T/D43B2.5 (•), and PLA/T/D43B5 (▲), into ethanol 10% (*v*/*v*).

Consequently, the release mechanism of thymol was evaluated by modelling the results obtained at different times (up to 15 days) in ethanol 10% (*v*/*v*). Figure 1a–c depicts the normalized plots obtained for the average amount of thymol released to the food simulant at a defined time, *M_F,t_*, by the amount of thymol released at the equilibrium at time t→∞, *M*_F,∞_, vs. time *t* (hours).

The quantitative assessment of *M_F,∞_* allowed the analysis of the diffusion process. For such purpose, Equation (1) was used to fit the experimental data. The corresponding data for *α* and *K_P,F_* (Table 1) were computed considering that *V_F_* was 20 cm^3^ of ethanol 10% (*v*/*v*) and the area of the PLA-based films used in these tests was 12 cm^2^.

According to the results shown in Table 1, two main conclusions can be obtained: (i) the cumulative amount of thymol released into ethanol 10% (*v*/*v*) decreased from 38% (without nanoclay) to 35 and 31% for 2.5 and 5 wt.% of D43B, respectively; (ii) the analysis of the partition coefficients (α and *K_P,F_*) showed their influence in the thymol diffusion mechanism. Therefore, it can be assumed that the release of thymol is governed by the Fick’s second law (Equation (2)) and the diffusion coefficients (*D*, cm^2^ s^−1^) were calculated (Table 1) from the least-square fit of Equation (5) to the experimental data (solid lines in Figure 1a–c). The RMSE (Equation (6)) of the experimental and estimated values between the calculated (*y_i_*) and observed (*ŷ_i_*) results for *M_F,t_/M_F,∞_* was minimized, providing a reliable indication of their fit, and it could be considered promising if the experimental error is taking into account the *M_F,t_/M_F,∞_* ratio. A good fit between calculated and experimental data was obtained for PLA/T and PLA/T/D34B2.5, with RMSE values of 0.0773 and 0.0698, respectively. However, a higher value was obtained for PLA/T/D43B5 (RMSE: 0.114), particularly for long-range times.

A more in-depth analysis of the fitting between experimental and calculated values showed that these results allow estimating the kinetics of thymol release at short times. Positive deviations of the fitting line for short times (i.e., *M_F,t_/M_F,∞_* < 0.60) and negative deviations for *M_F,t_/M_F,∞_* > 0.60 were observed in all cases. For such purpose, Equation (8) can be used for a linear regression analysis and as a simplified release model derived from Equation (5) [41]:

(8) $${\left[\frac{1}{\pi }-\frac{1}{\alpha }\cdot \frac{M_{F,t}}{M_{P,0}}\right]}^{0.5}=-\frac{D^{’0.5}}{\alpha \cdot l}{\cdot t}^{0.5}+\frac{1}{\pi^{0.5}}$$

Diffusion coefficients for short times, (*D’*, cm^2^ s^−1^), were computed by using the linear fitting of Equation (8) to the experimental data (Figure 1d). Results obtained showed a very good fit between computed and experimental values for the first term in Equation (8) as a function of ***t^0,5^***, with coefficients of determination (*R*^2^) higher than 0.999, suggesting that the experimental release data are well described by the proposed diffusion model for short-range times.

However, it was observed that the total data range cannot be fully characterized by a Fickian diffusion process, probably due to the lack of fitting caused by the last points in the plot resulting in poor results in the fitting to the first data, called short-range time. However, it was also observed that better fitting values were obtained after application of Equation (8). Therefore, the discrepancy in *D* values obtained (*D* and *D’*) from Equation (5) and Equation (8) was a good indication that a non-Fickian release model was observed in this system. The failure of the Fickian solution process to predict the release kinetics may be due to the polymer matrix’s structural modifications caused by the progressive deterioration of the PLA matrix due to the direct contact with ethanol, which can act as a plasticizer in this system. This could result in the erosion of the polymer by opening the PLA’s internal structure, creating interstitial spaces that could favor the release of thymol over time due to the concentration gradient [42,43]. Moreover, as the diffusion rate increased (*D* > *D’*), the intermolecular interactions between ethanol molecules and PLA chains were enhanced at long times [44]. No reports on the diffusion coefficient for thymol in nanocomposite films based on PLA have been published, but higher values than those obtained in this study were reported in polypropylene (PP) films using ethanol 10% (*v*/*v*) as food simulant (1.75 × 10^−10^ cm^2^ s^−1^) [45]. Torres et al. [46] evaluated the thymol release from LDPE films in ethanol 10% (*v*/*v*). They indicated that the diffusion coefficient values ranged from 7.5 × 10^−8^ to 1.8 ×10^−8^ cm^2^ s^−1^, which were higher than those obtained in this work. These differences could be mainly due to the lower density and linear structure of LDPE compared to PLA, resulting in higher mass transport properties.

*D* values calculated in this work are consistent with the thymol release profiles shown in Figure 1a–c. The presence of D43B led to a decrease in the thymol release during the study at 40 °C, which was consistent with our previous study showing a shift in the XRD peak of D43B, suggesting the intercalated morphology of the PLA-D43B nanocomposites. In this case, a shift of the clay diffraction peak to lower angles of 2θ = 4.6° was observed, corresponding to an interlayer distance of 35.6 Å [30]. Moreover, Campos-Requena et al. [37] reported that the influence of the chemical modification of clays on the active compound profile release could be a factor in the modelling of an active packaging system with controlled release of volatile compounds.

### 3.2. Antibacterial Activity

The antibacterial activity of the developed active nano-biocomposite films was evaluated against Gram-positive (*S. aureus* 8325-4) and Gram-negative bacteria (*E. coli*) by using the direct contact method. Table 2 shows the cell viability related to neat PLA, expressed as the percentage of microorganisms proliferated onto the PLA-based films after 3 and 24 h incubated at 4, 24 (room temperature), and 37 °C, respectively. Significant differences in cell viability for both bacterial strains were observed for almost all formulations compared to neat PLA at the tested experimental conditions. Exciting features could be observed when comparing the results obtained for active formulations containing thymol (PLA/T, PLA/T/D43B2.5, and PLA/T/D43B5) and their non-active counterparts (PLA/D43B2.5 and PLA/D43B5). These results are in accordance with those reported by Liu et al. [47] who found that the antibacterial activity of phenolic monoterpenes, including thymol, is related to their ability to be released through the polymer matrix over time allowing their continuous availability and diffusion through the bacterial cell membrane. Thymol can attach to the cell surface, and thereafter, penetrate the phospholipid bilayer of the cell membrane. The relative position of the hydroxyl group is crucial for the bioactivity of thymol, which explains its superior antimicrobial action as compared to other plant phenolics, such as 2-amino-p-cymene, which has a similar structure than thymol except for the hydroxyl group [48].

PLA formulations with only D43B, without the presence of thymol, also showed antibacterial activity against *E.* coli and S. aureus 8325-4 strains at both incubation times and all tested temperatures (4, 24 and 37 °C). PLA/D43B2.5 and PLA/D43B5 showed similar cell viability in most of the tested conditions. The only set of experimental conditions where PLA/D43B2.5 showed significantly higher antimicrobial activity than PLA/D43B5 was at 4 °C and 24 h for S. aureus 8325-4, with values 88.6 ± 0.9 and 94.4 ± 1.7%, respectively. These results are in agreement with those reported by De Azeredo et al. [49], who concluded that organo-modified montmorillonites could also produce the rupture of cell membranes by themselves, resulting in the inactivation of both Gram-positive and Gram-negative bacteria. This effect was attributed to the presence of quaternary ammonium groups able to react with lipids and proteins in the microorganism cell wall. Hong and Rhim [50] proved that organically modified clay powders with a quaternary ammonium salt, such as D43B, possess strong antimicrobial activity against *S. aureus*, *Listeria monocytogenes*, *Salmonella typhimurium,* and *E. coli* O157:H7.

The obtained antibacterial activity in the ternary systems resulted much more efficient and statistically significant with reference to the PLA control film. The percentage of bacteria viability was lower for PLA/T/D43B2.5 and PLA/T/D43B5 incubated with *S. aureus* 8325-4 strains (44.3 ± 1.4% and 48.5 ± 1.1%, respectively) and *E. coli* strains (47.2 ± 1.2% and 47.3 ± 1.4%, respectively) for 3 h at 37 °C (Table 2). Regarding incubation time, the percentage of the surviving fraction of bacteria did not show significant differences (*p* > 0.05) between 3 and 24 h for both bacterial strains, confirming the bacteriostatic action of the PLA-based nanocomposites. Concerning incubation temperature, the percentage of cell viability for both bacterial strains was lower when incubating at 37 °C whereas it was moderately increased at 4 °C. These results are in line with the thymol release data, thus confirming that the temperature may influence the diffusion and swelling properties of the PLA matrix, promoting the diffusion of thymol through the biopolymer structure.

### 3.3. Disintegrability Under Composting Conditions

Table 3 shows the values of weight loss of each sample and Table 4 shows the visual appearance of nano-biocomposite films at different times under composting conditions. It was observed that after 4 days, the disintegration rate of PLA-based materials increased significantly for binary and ternary systems showing an evident fragmentation, reaching all materials a degree of disintegration exceeding 90% after 35 days. In fact, after 4 days, all samples changed their visual appearance with a general whitening effect, loss of transparency, and evident deformation and size reduction. These results are indicative of the beginning of the hydrolytic degradation process caused by simultaneous changes in the refractive index due to water absorption, with the formation of low molecular weight by-products and the resulting increase in the PLA crystallinity [51].

According to Su et al. [52] this first step, covering the first 5 days of this study where the weight loss was small, corresponds to slow bulk degradation processes with a surface-erosion mechanism that can be mainly caused by hydrolysis, resulting in small molecules (mostly water) that can diffuse through the polymer matrix. The diffusion rate is influenced by several factors, such as crystallinity, cross-linking degree, and other morphological properties. After 5 days, the weight loss increased dramatically reaching values higher than 40% after 7 days and a continuous increase with time up to 35 days when more than 90% of the initial weight was lost (Table 3). In that period, the hydrolysis reaction was still important, and the average molecular weight of PLA decreased continuously forming small fragments easier to disintegrate since the internal chains were increasingly exposed. In that period, water, compost, and the microbiota generated in the reactor can penetrate into the gaps in the PLA structure formed by the hydrolysis reactions, contributing to a clear acceleration of the disintegration process which causes visual modifications by the formation of small particles, fragmentation and irreversible changes in mechanical properties.

The use of nanoclays, such as D43B, and thymol as active additive influenced the disintegration rate in compost of PLA since this process is strongly dependent on the hydrophilic/hydrophobic character of the nanocomposite and this parameter changes due to the presence of hydroxyl groups from thymol and the organic modifier of D43B [53]. Hydroxyl groups can contribute to the heterogeneous hydrolysis of PLA by absorbing water from the medium resulting in a noticeable formation of labile bonds in the PLA structure with the consequence of a significantly higher disintegration rate [54]. It was also observed that binary and ternary systems suffered physical breakage with a considerable increase in weight loss (Table 4) after 7 days, showing significant differences in the disintegration profile when comparing neat PLA and nano-biocomposite films. Results at longer times showed that physical degradation progressed with burial time, resulting in the complete disintegration of all the initial samples after 35 days, when the disintegration degree exceeded 90% covering the ISO 20,200 requirements (Table 3).

**Figure 2:**
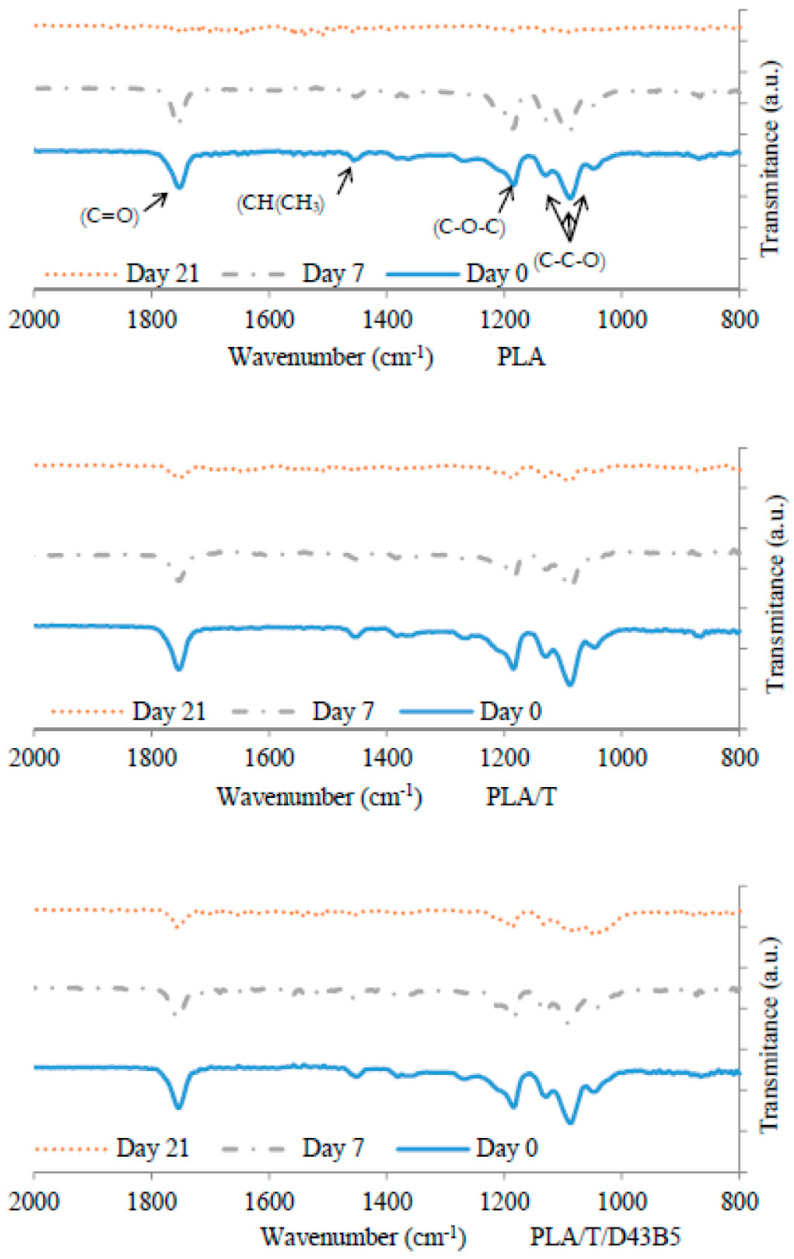
FTIR spectra of PLA, PLA/T, and PLA/T/D43B5 before (0 days) and after different incubation times (7 and 21 days) under composting conditions.

FTIR analysis of neat PLA and nano-biocomposite films at different times was carried out to evaluate the structural changes produced by the disintegration process in all formulations. Figure 2 shows the FTIR spectra obtained for neat PLA, PLA/T and PLA/T/D43B5 after 0, 7 and 21 days under composting conditions. PLA showed characteristic bands at 1750 cm^−1^ (C = O), 1440 cm^−1^ (CH–CH_3_), and 1267 cm^−1^(C–O–C) as well as three peaks at 1123, 1082 and 1055 cm^−1^ related to the C–C–O groups. After 7 days, the intensity of the three peaks related to the C–C–O groups decreased and after 21 days, these peaks disappeared for all formulations. Similar results were obtained by Fortunati et al. [53] who proposed that the modification of the intensity of peaks related to the C–C–O groups can be associated with the scission of the PLA interchain bonds produced by hydrolysis reactions occurring during disintegration tests. Moreover, the C–O–C stretching vibration at 1267 cm^−1^ was also affected by the depletion of the lactic acid and oligomer molecules caused by microorganisms, leaving highly reactive carboxylate end groups [55]. FTIR results agreed with those achieved for the disintegration weight loss and with the progressive disintegration of all samples with increasing testing time.

**Figure 3:**
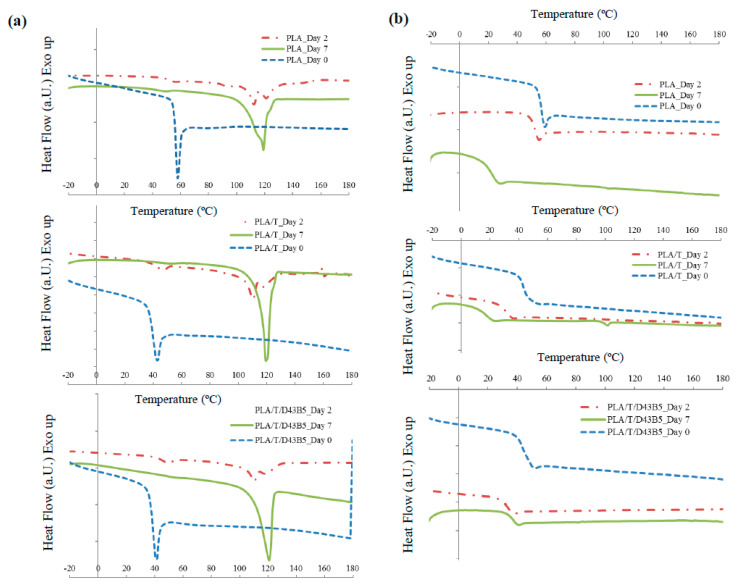
DSC thermograms, 1st heating scan (**a**) and 2nd heating scan (**b**), of PLA-based nano-biocomposite films after different composting times.

Figure 3a shows an example of the DSC thermograms obtained from the first heating scan for PLA, PLA/T and PLA/T/D43B5 films at different composting times. The endothermic peak observed immediately after the T_g_ at day 0 for all the tested materials corresponds to the enthalpic relaxation process. This effect was related to the aging process of PLA, which was previously observed by other authors [56]. However, the initially amorphous PLA-based materials developed multiple endothermic peaks just after 7 days under composting conditions. In fact, the enthalpic relaxation peak gradually disappeared due to the hydrolysis process at short incubation times. Yang et al. [57] related this behavior to the moisture absorption happening under composting conditions, since water could serve as a plasticizer agent in PLA matrices. These effects could also be related to the well-dispersed nanofiller inside the polymer matrix. Olewnik-Kruszkowska et al. [58] associated the hydrolytic degradation to the decrease in all thermal properties and they confirmed that the highest changes in the T_g_ value could be related to the dispersion of the nanofiller inside the polymer matrix.

The gradual disintegration suffered when increasing the testing time resulted in the observation of new melting peaks related to the formation of crystalline structures with different perfection degrees in the PLA matrix (Figure 3a). These results were found for all samples and they can be correlated with the observed visual changes, since hydrolysis promotes crystallization in the polymer matrix, resulting in significantly essential changes in the visual appearance of the testing samples and their disintegrability behavior. Similar results were reported by other authors, who suggested that the appearance of multiple melting peaks could be related to the formation of different crystal structures due to the polymer chain scission produced during degradation [59,60].

Figure 3b shows an example of the DSC thermograms recorded during the second heating scan for PLA, PLA/T and PLA/T/D43B5 samples submitted to the disintegration test. It was observed that after 2 days, all PLA-based films showed a significant decrease in T_g_. Previous work by our research group showed that this decrease in T_g_ values was due to the increase in the mobility of the polymer chains as a consequence of the hydrolytic process [55] and the formation of lactic acid oligomers and low molecular weight by-products with a plasticizing effect in the polymer structure and the consequent changes in their visual appearance.

## 4. Conclusions

The incorporation of thymol, as active additive, and D43B, as nano-reinforcing agent, into PLA has shown as an accessible and useful route for the preparation and modification of PLA nano-biocomposite films properties. An improvement in functional properties of PLA-based films was obtained due to the addition of the active additive and the nanoclay resulting in enhanced antimicrobial and antioxidant properties, demonstrating the high potential of the developed formulations for food packaging applications without compromising the inherent biodegradation properties of the PLA matrix. The obtained results suggest the possibility of controlling the release of active additives in the design of active nano-biocomposite films through the incorporation of laminar nanoclays by the decrease in the diffusion of thymol through the polymer matrix by the formation of tortuous paths. The antibacterial activity of these active nanocomposites was proved against two different bacterial strains, showing the PLA/T/D43B2.5 formulation the best results against both *S. aureus 8325-4* (44.3 ± 1.4%) and *E. coli* (47.2 ± 1.2%) at 37 °C and 3 h of incubation time. From a practical point of view, the combination of 8 wt.% of thymol and 2.5 wt.% of D43B added into a commercial PLA matrix showed high potential for the development of new bio-based and biodegradable active packaging films with application in prolonging the shelf-life of fresh food.

## Figures and Tables

**Table 1:** Parameters obtained for the release of thymol from PLA-based films into ethanol 10% (*v*/*v*).

	PLA/T	PLA/T/D43B2.5	PLA/T/D43B5
M_P,0_ (mg)	16.60 ± 0.20	17.82 ± 0.09	17.21 ± 0.05
M_F,∞_ (mg)	6.25 ± 0.22	6.31 ± 0.27	5.29 ± 0.29
l/cm	0.0167	0.0215	0.0180
A	1.65	1.82	2.25
K_P,F_	60.3	42.4	41.1
**Equations (5) and (6):**			
D (cm^2^ s^−1^)	3.36 × 10^−11^	4.86 × 10^−11^	2.25 × 10^−11^
RMSE	0.0773	0.0698	0.114
**Equations (6) and (8):**			
D’ (cm^2^ s^−1^)	5.95 × 10^−12^	7.45 × 10^−12^	5.82 × 10^−12^
RMSE	0.00362	0.00306	0.00370

**Table 2:** Antibacterial activity of PLA-based nano-biocomposite films obtained at 2 incubation times (3 h and 24 h) and 3 different temperatures (4, 24 and 37 °C) against *E. coli* and *S. aureus* 8325-4, expressed as cell viability (%).

Formulation	*Cell Viability (%)*			
	*S. Aureus* 8325-4		*E. Coli*	
	3 h	24 h	3 h	24 h
**At 4 °C**				
PLA	100.0 ± 1.7 ^a^	100.0 ± 1.8 ^a^	100.0 ± 6.7 ^a^	100.0 ± 2.5 ^a^
PLA/T	83.5 ± 3.3 ^b^	85.5 ± 1.1 ^c^	65.4 ± 5.4 ^b^	60.6 ± 1.0 ^c^
PLA/D43B2.5	98.0 ± 1.1 ^a^	88.6 ± 0.9 ^b,c^	83.5 ± 2.9 ^a^	74.0 ± 4.2 ^b^
PLA/D43B5	91.1 ± 1.3 ^c^	94.4 ± 1.7 ^a,b^	88.0 ± 3.2 ^a^	77.8 ± 3.5 ^b^
PLA/T/D43B2.5	68.0 ± 0.3 ^d^	60.9 ± 2.0 ^d^	53.7 ± 3.3 ^b^	52.0 ± 0.5 ^c,d^
PLA//T/D43B5	70.0 ± 0.5 ^d^	61.6 ± 1.0 ^d^	54.4 ± 2.9 ^b^	50.0 ± 0.7 ^d^
**At 24 °C**				
PLA	100.0 ± 1.0 ^a^	100.0 ± 0.5 ^a^	100.0 ± 3.8 ^a^	100.0 ± 2.2 ^a^
PLA/T	72.8 ± 2.2 ^b^	62.4 ± 1.5 ^b^	69.5 ± 1.6 ^b^	66.2 ± 1.4 ^c^
PLA/D43B2.5	83.4 ± 1.1 ^a^	83.4 ± 1.4 ^c^	75.3 ± 2.8 ^b^	82.6 ± 1.9 ^b^
PLA/D43B5	75.4 ± 1.4 ^c^	81.2 ± 1.7 ^c^	70.3 ± 1.3 ^b^	80.0 ± 3.3 ^b^
PLA/T/D43B2.5	56.1 ± 1.2 ^d^	59.4 ± 1.4 ^b^	53.1 ± 0.2 ^c^	57.9 ± 0.7 ^c,d^
PLA//T/D43B5	56.6 ± 2.0 ^d^	58.7 ± 0.8 ^b^	59.6 ± 2.0 ^c^	60.2 ± 1.5 ^d^
**At 37 °C**				
PLA	100.0 ± 3.8 ^a^	100.0 ± 2.1 ^a^	100.0 ± 1.7 ^a^	100.0 ± 3.1 ^a^
PLA/T	57.9 ± 2.2 ^b^	73.3 ± 1.2 ^c^	62.9 ± 0.5 ^b^	71.2 ± 1.4 ^c^
PLA/D43B2.5	63.8 ± 2.0 ^b^	84.6 ± 0.9 ^b^	67.6 ± 0.9 ^b^	79.4 ± 1.2 ^b^
PLA/D43B5	64.3 ± 1.5 ^b^	89.8 ± 2.0 ^b^	64.8 ± 2.3 ^b^	71.7 ± 1.2 ^c^
PLA/T/D43B2.5	44.3 ± 1.4 ^c^	51.6 ± 0.5 ^d^	47.2 ± 1.2 ^c^	49.3 ± 0.5 ^d^
PLA/T/D43B5	48.5 ± 1.1 ^c^	59.6 ± 3.2 ^e^	47.3 ± 1.4 ^c^	53.2 ± 1.2 ^d^

Data are expressed as cell viability (%), which corresponds to the percentage of the CFU of bacteria growth in nano-biocomposite films compared to that obtained in PLA (set as 100%). Results are expressed as mean ± standard deviation, *n* = 3. Different superscripts (a, b, c, d, and e) within the same column at a specific temperature indicate statistically significant different values compared at 3 and 24 h for *S. aureus* 8325-4 and *E. coli* at different temperatures.

**Table 3:** Disintegrability values (mean ± standard deviation, *n* = 3, %) of PLA and nano-biocomposite films at different times under composting conditions. Different superscripts (a, b, c, and d) within the same row indicate statistically significant different values.

Time (days)	Disintegrability (%)					
	PLA	PLA/T	PLA/T/D43B2.5	PLA/T/D43B5	PLA/D43B2.5	PLA/D43B5
2	0.3 ± 0.1 ^a^	5.1 ± 0.4 ^c^	8.4 ± 0.9 ^d^	7.2 ± 0.3 ^d^	2.4 ± 0.7 ^b^	1.2 ± 0.1 ^a,b^
4	0.4 ± 0.1 ^a^	3.5 ± 0.2 ^b,c^	5.2 ± 1.2 ^c,d^	6.0 ± 0.2 ^d^	2.3 ± 0.2 ^a,b^	2.0 ± 0.1 ^a,b^
7	42.2 ± 3.8 ^a^	51.3 ± 0.2 ^b^	57.9 ± 2.3 ^b^	49.8 ± 0.9 ^b^	53.0 ± 2.2 ^b^	54.5 ± 1.8 ^b^
10	56.3 ± 4.6 ^a^	72.0 ± 4.3 ^b^	76.0 ± 1.3 ^b^	77.3 ± 4.9 ^b^	67.8 ± 1.5 ^b^	69.9 ± 4.3 ^b^
14	72.4 ± 2.8 ^a^	65.6 ± 3.3 ^a^	68.9 ± 3.9 ^a^	65.5 ± 4.5 ^a^	64.9 ± 6.4 ^a^	64.0 ± 3.8 ^a^
21	73.2 ± 6.7 ^a^	79.5 ± 2.4 ^a^	82.4 ± 3.4 ^a^	81.8 ± 2.8 ^a^	82.2 ± 4.3 ^a^	78.2 ± 1.6 ^a^
28	77.3 ± 1.4 ^a^	81.9 ± 1.6 ^a^	82.2 ± 0.8 ^a^	79.7 ± 6.8 ^a^	76.2 ± 1.6 ^a^	77.5 ± 4.9 ^a^
35	95.7 ± 0.7 ^a^	98.0 ± 0.5 ^a^	95.5 ± 0.9 ^a^	97.8 ± 0.5 ^a^	97.2 ± 1.2 ^a^	96.5 ± 1.7 ^a^

**Table 4:** Visual appearance of PLA and nano-biocomposite films at different times under composting conditions. Different superscripts within the same column indicate statistically significant different values.

Formulation	Time (Days)							
	0	2	4	7	10	14	21	28
PLA	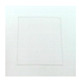	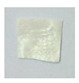	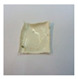	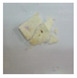	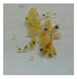	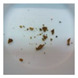	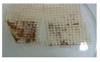	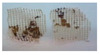
PLA/T	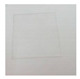	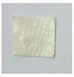	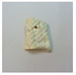	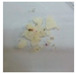	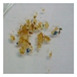	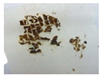	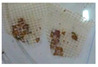	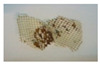
PLA/T/D43B2.5	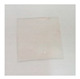	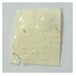	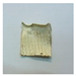	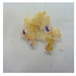	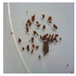	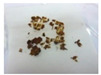	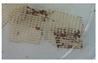	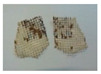
PLA/T/D43B5	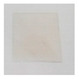	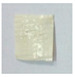	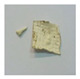	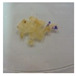	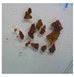	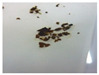	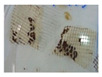	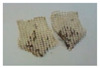
PLA/D43B2.5	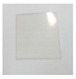	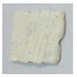	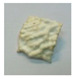	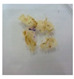	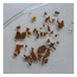	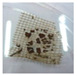	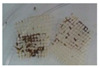	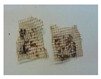
PLA/D43B5	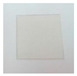	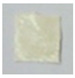	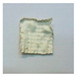	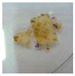	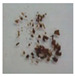	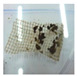